# First Experiences with Fusion of PET-CT and MRI Datasets for Navigation-Assisted Percutaneous Biopsies for Primary and Metastatic Bone Tumors

**DOI:** 10.3390/diagnostics15010063

**Published:** 2024-12-29

**Authors:** Hagen Fritzsche, Alexander Pape, Klaus-Dieter Schaser, Franziska Beyer, Verena Plodeck, Ralf-Thorsten Hoffmann, Patricia Hahlbohm, Elisabeth Mehnert, Anne Weidlich

**Affiliations:** 1 University Center for Orthopedics, Trauma Surgery and Plastic Surgery, University Hospital Carl Gustav Carus Dresden, 01307 Dresden, Germany; alexander.pape@ukdd.de (A.P.); klaus-dieter.schaser@ukdd.de (K.-D.S.); anne.weidlich@ukdd.de (A.W.); 2 AG Knochentumoren e.V., CH-4031 Basel, Switzerland; 3 Sarcoma Center at the National Center for Tumor Diseases (NCT/UCC), University Hospital Carl Gustav Carus Dresden, 01307 Dresden, Germany; 4 Clinical Epidemiology at University Center for Orthopedics, Trauma Surgery and Plastic Surgery, University Hospital Carl Gustav Carus Dresden, 01307 Dresden, Germany; franziska.beyer@ukdd.de; 5 Institute for Diagnostic and Interventional Radiology, University Hospital Carl Gustav Carus Dresden, 01307 Dresden, Germany; verena.plodeck@ukdd.de (V.P.); ralf-thorsten.hoffmann@ukdd.de (R.-T.H.); patricia.hahlbohm@ukdd.de (P.H.)

**Keywords:** navigation, PET-CT, MR imaging, tumor biopsy, bone tumors, radiation exposure

## Abstract

**Background:** The aim of this study was to compare the technique of navigation-assisted biopsy based on fused PET and MRI datasets to CT-guided biopsies in terms of the duration of the procedure, radiation dose, complication rate, and accuracy of the biopsy, particularly in anatomically complex regions. **Methods:** Between 2019 and 2022, retrospectively collected data included all navigated biopsies and CT-guided biopsies of suspected primary bone tumors or solitary metastases. Navigation was based on preoperative CT, PET-CT/-MRI, and MRI datasets, and tumor biopsies were performed using intraoperative 3D imaging combined with a navigation system. **Results:** A total of 22 navigated (main group: m/f = 10/12, mean age: 56 yrs.) and 57 CT-guided biopsies (reference group: m/f = 36/21, mean age: 63 yrs.) were performed. Patients were grouped according to anatomic sites (pelvis, spine, extremities, thorax). The duration of the procedure in the reference group was significantly shorter than in the main group, particularly in the spine. The effective radiation dose was in the same range in both groups (main/reference group: 0.579 mSv and 0.687 mSv, respectively). In the reference group, a re-biopsy had to be performed in nine patients (diagnostic yield: 84%). A total of four major and three minor complications occurred in the reference group. **Conclusions:** Navigation-assisted percutaneous tumor biopsy resulted in correct, histologically useable diagnoses in all patients and reached a higher accuracy and first-time success rate (diagnostic yield: 100%) in comparison to CT-guided biopsies. The fusion of PET, CT, and MRI datasets enables us to combine anatomical with metabolic information. Consequently, target selection was improved, and the rate of false negative/low-grade sampling errors was decreased. Radiation exposure could be kept at a comparable level, and the durations of both procedures were comparable to conventional methods.

## 1. Introduction

Among the factors that initially determine the adequate management of skeletal tumors, an accurate biopsy followed by a comprehensive histopathological assessment is essential and the most important step towards correct diagnosis. However, to ensure a reliable histopathological diagnosis, high-quality tissue sampling from high-grade tumor areas with maximum contrast medium enhancement in the MRI or metabolic activity as shown by FDG-PET uptake is of crucial importance [1,2]. Current biopsy techniques for bone tumors typically include either open incisions controlled simply by conventional radiographic imaging (C-arm device) or CT-guided needle biopsies. Concerning open biopsies, the intraoperative identification and localization of the lesion, as well as sufficient sampling of the high-grade part of the lesion under simple image converter control, however, are very difficult or even impossible due to the often small size, variable radiation density, and heterogeneous structure of the lesion and the lack of any information reflecting contrast medium enhancement or metabolic activity. For this reason, intraoperative orientation is based on preoperative MRI to differentiate between hematoma, necrosis, and contrast medium-enhanced areas, presumably reflecting low- or high-grade areas. Recent studies have shown that, in CT-guided biopsies [3,4,5,6,7,8,9] and open biopsies [10], the lesion is sometimes not reliably targeted, as either low-grade hematoma or necrotic tissue is erroneously detected. Therefore, CT-guided biopsies often show a lower diagnostic yield, so navigated biopsies are expected to provide better results [11,12,13,14,15].

Apart from CT-guided investigations, studies that apply the navigation technique for diagnostic biopsy, particularly those that use a fusion of CT, MRI, and PET datasets for optimized visualization and percutaneous targeted biopsy of selected high-grade parts of the tumor, are missing.

Therefore, this study aimed to apply and use a fusion of datasets of CT, MRI, and PET for navigated biopsies. The practical experiences and results should be analyzed in terms of a description of the navigation technique using fused datasets, general feasibility, duration/time of the operation, radiation dose, and complication rates, as well as the accuracy of the biopsy. Also, the navigated technique should be compared to CT-guided biopsies.

## 2. Materials and Methods

Patient data from January 2019 to December 2022 were collected retrospectively for all cases in which a biopsy of a suspected primary bone tumor or solitary metastasis in anatomically complex regions was performed. The navigated biopsies were defined as the main group and the CT-guided biopsies as the reference group. The investigation was approved by our institutional review board (BO-EK-189052020) and was carried out in accordance with the latest version of the Declaration of Helsinki.

The same team of musculoskeletal surgeons (*n* = 2, with more than 10 years of experience) performed all navigated tumor biopsies. The indication for navigation-assisted biopsy technique was based on the following criteria:

- Very small tumors (size <1–2 cm);
- Tumors located in anatomically complex regions, which were therefore difficult to reach via a conventional biopsy technique (spine, pelvis, sacrum);
- Tumors only visible on MRI and not CT;
- Tumors that displayed very heterogeneous FDG-uptake patterns in PET-CT and heterogeneous enhancement on MRI, indicative of either variable intratumoral differentiation (low- and high-grade parts) or necrosis/hematoma formation.

All patients included in the main group underwent preoperative diagnostic imaging, i.e., conventional radiographs followed by FDG-PET-CT and MRI scans (different MRI scanners; protocols individually adapted to tumor location, generally including T2-weighted sequences, T1-weighted sequences, and contrast-enhanced T1-weighted sequences with fat saturation). PET–CT imaging was performed on a Siemens Biograph 16. The SUVmax (standardized uptake value) was determined for FDG-avid focus sites. PET-MRI was performed on a 3 Tesla Ingenuity TOF PET/MRI (Philips Medical Systems, Best, the Netherlands).

Based on their radiographic appearance in the CT scan and as assessed by MRI findings all bone lesions were quantitatively analyzed concerning their tissue type (osteolytic, osteoblastic, mixed) in order to explore its effect on the diagnostic yield of both procedures (CT-guided vs. navigated biopsy).

**Figure 1:**
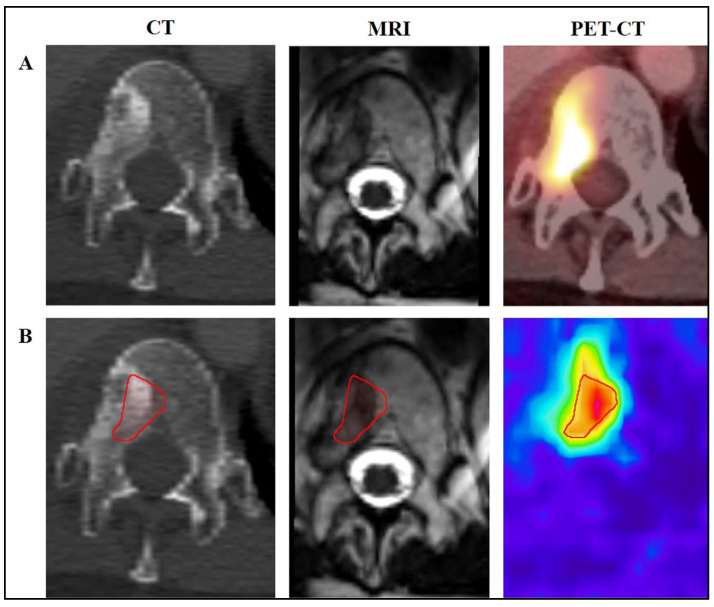
A 54-year-old patient with a recurrence of dedifferentiated (G3) chondrosarcoma at the level of the thoracic spine (Th 12). (**A**) Preoperative imaging as part of the staging including CT, MRI, and PET-CT. (**B**) Preoperative fusion of CT imaging, MR imaging, and PET-CT and marking of the tumor in its total size or, as in this example, marking of the region with the highest activity in PET-CT using Smartbrush software.

As a first step, the preoperative CT, MRI, and PET data were merged, and the tumor area/volume in all planes was then marked in red for better recognition using Smartbrush software (Brainlab AG, Munich, Germany, version 5.0.0.165). Hyperbolic FDG-avid foci (SUVmax-sites) on PET-CT/-MRI can be marked as the primary target for biopsy procedure/trocar placement. Fusion of images was performed by image processing using the software provided by the Image Guided Navigation system (Curve™, Brainlab AG, Munich, Germany, Figure 1).

**Figure 2:**
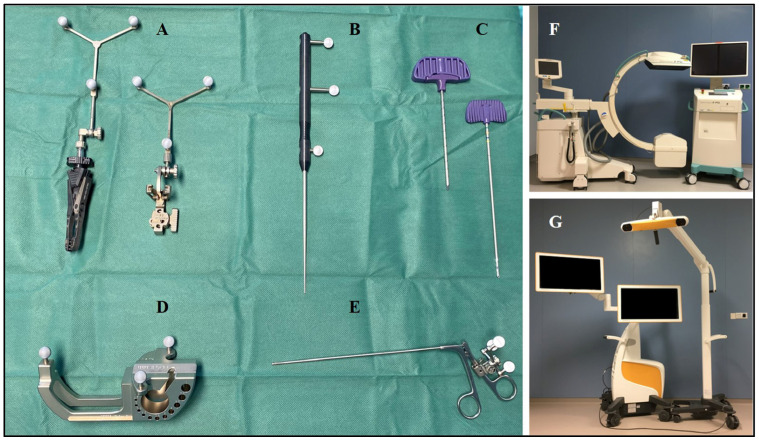
Intraoperative instruments and devices for navigated biopsies. (**A**) Reference basis frame (tracker). (**B**) Standard needle probe (pointer). (**C**) Bone-access instrument (KyphonTM Xpander II Osteo Introducer, size 3.8 ga access, Medtronic). (**D**) Calibration instrument. (**E**) Rongeur. (**F**) Three-dimensional flat panel C-arm (Ziehm Vision RFD). (**G**) Optoelectronic navigation system (Curve™ Image Guided Navigation, Brainlab).

During the surgery, the patient was placed on a radiolucent carbon operating table. The surgical area was then covered using a sterile and removable drape and an intraoperative 3D scan (DVT, digital volume tomography using Ziehm Vision RFD, Figure 2) was performed with the reformation of axial, coronal, and sagittal views (3D dataset).

**Figure 3:**
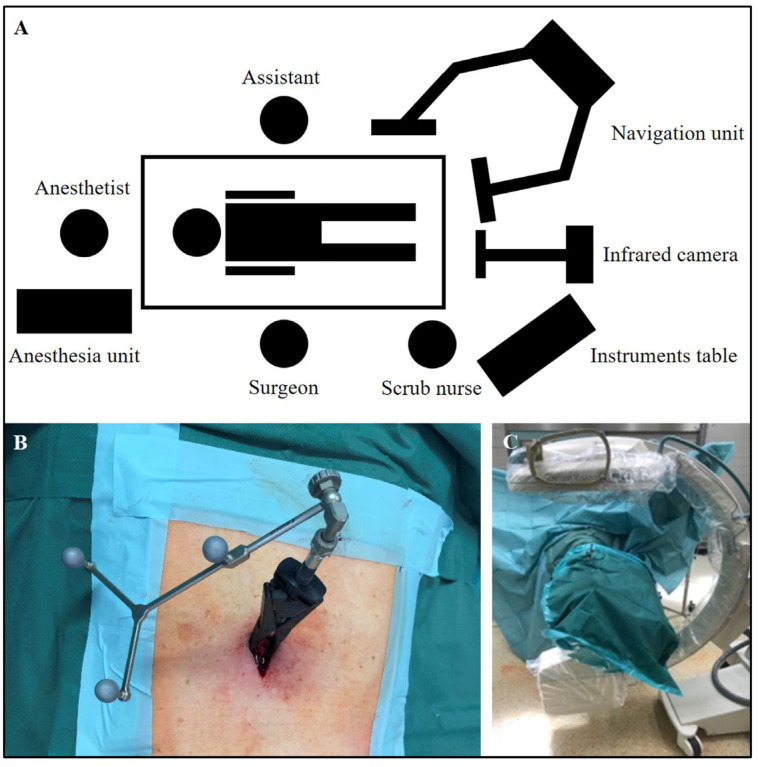
Intraoperative application and implementation of the navigation technique. (**A**) Set-up for the navigation, arrangement of persons, and positioning of the patient in the OR during navigation from a bird’s-eye view. (**B**) Attachment of the reference base by carbon clamp on the upper lumbar spine (L1) of a 54-year-old patient with a recurrence of chondrosarcoma at the level of the thoracic spine (Th 12). (**C**) Positioning of the C-arm and covering the patient.

**Figure 4:**
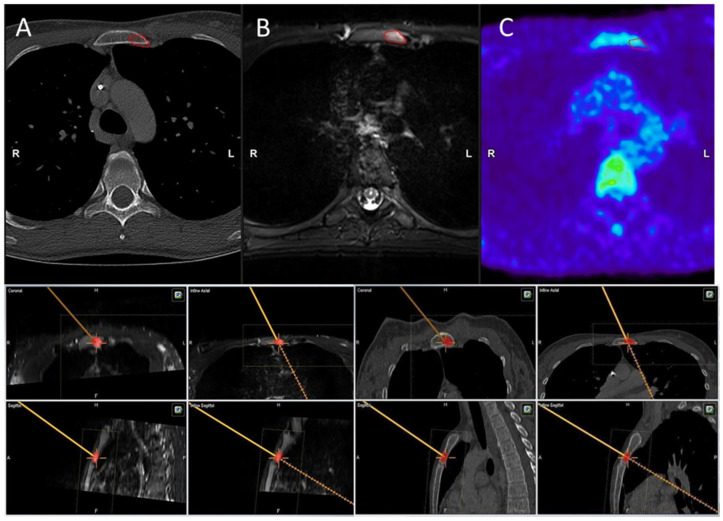
A 35-year-old patient with a solitary metastasis in the manubrium sterni with breast carcinoma. The tumor was not visible in the CT scan (**A**), only in MRI (**B**) and PET-CT (**C**).

**Figure 5:**
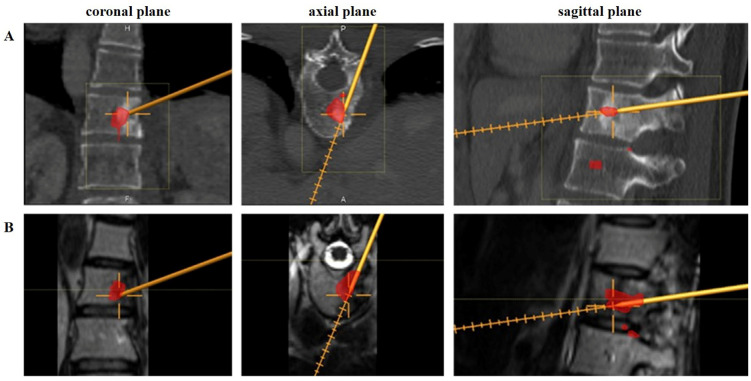
A 54-year-old patient with a recurrence of chondrosarcoma at the level of the thoracic spine (Th 12). The area corresponding to the FDG-avid lesion in the thoracic spine (Th 12) was marked with Smartbrush software for navigated biopsy, as visualized in (**A**) CT and (**B**) MR imaging.

As all navigated biopsies were percutaneously planned, the exposure of anatomic skeletal landmarks for surface matching with diagnostic CT scans was not needed as an intraoperative 3D scan was performed. Prior to that, a reference base clamp (navigation tracker) was installed using 3.0 mm pins, or a carbon clamp was attached to skeletal segment/cortical bone near the operating area via small stab incisions. The acquired intraoperative 3D-scan data were precisely merged with the preoperative diagnostic CT, MRI, and PET data to create the virtual image dataset used for further navigation. Thus, multiplanar CT and MR imaging could be visualized simultaneously (Figure 3 and Figure 4). To plan the biopsy according to established guidelines [16], a probe with a mounted navigation tracker was used as a pointer tool (Brainlab AG, Munich, Germany). This tool, with a variable offset function, enabled the three-dimensional determination of the biopsy direction and the virtual projection of the planned biopsy tract directly to the high-grade biopsy target zone (SUVmax focus) (Figure 2). Subsequently, a 3 mm stab incision was made, through which the tracked pointer tool marked the cortical entry point. A bone-access cannula (KyphonTM Xpander II Osteo Introducer, size 3.8 ga access, Medtronic, Figure 2) was then placed, facilitating the insertion of a navigated rongeur for the final biopsy (Figure 5).

Successful biopsy was assessed through immediate intraoperative frozen section analysis, confirming the sampling of a sufficient amount of viable tumor tissue as well as the definitive histopathology (tumor entity). For all sarcoma patients, the histopathological results regarding differentiation/grading of the biopsy and final resection were compared, and any downgrading or upgrading was evaluated.

The dose area products were measured using the C-arm devices. Based on the focus-to-detector and the focus-to-patient distances, the entrance dose was calculated by applying the inverse square law to convert the image area to the irradiated area on the patient surface. The dose area product was divided by this area to determine the entrance dose, which was then converted to organ doses using conversion factors [17]. From the organ doses, the effective radiation dose was calculated by applying tissue weighting factors [18].

CT-guided biopsies were performed using the Bedford^TM^ Guided Hard Bone Biopsy System (11G) or Arrow^®^ OnControl^®^ Powered Bone Access System (11/13G). Biopsies were performed under analgosedation and local anesthesia with Xylocitin. Patients were positioned within the CT scanner (Siemens Somatom Definition AS+) depending on the location of the target lesion. A non-contrast-enhanced planning scan with a slice thickness of 3 mm was performed. The optimal access route was planned based on the localization of the lesion. The biopsy was performed under sterile conditions, and the specimens were collected in jars containing formaldehyde and sent for histopathologic analysis.

- Bedford^TM^ Guided Hard Bone Biopsy System:

Following a skin incision, the guide rod was positioned under CT fluoroscopy and anchored into the cortical bone. The perforating cannula was then placed over the rod and rotated counterclockwise through the soft tissue. Subsequently, the rod was replaced by a drill, which was locked together with the cannula. After the clockwise insertion of the biopsy needle, at least one tissue sample was successfully retrieved.

- Arrow^®^ OnControl^®^ Powered Bone Access System:

Under CT fluoroscopy, the biopsy needle, consisting of an outer cannula and inner stylet, was advanced to the bone periosteum. After the powered driver drill was attached, the needle was advanced through the cortical bone. The inner stylet was then removed, and the outer cannula was further advanced through the lesion using the drill. Once the biopsy was completed, the outer cannula was extracted while keeping the drill mode activated.

The data were evaluated in terms of epidemiology, radiation dose, duration of surgery, complications, and accuracy of biopsy. Continuous values are presented as median (quartiles), while categorial values are given as absolute and relative frequencies. Group comparisons were performed using the Mann–Whitney U-Test for continuous values and the chi-square test for categorical values, with a significant level set at *p* < 0.05. The software SPSS (version 28 for Windows) was used for data analysis.

Complications were categorized as major and minor. Major complications included postoperative cerebral hemorrhage, pneumothorax, significant bleeding, aspiration, postoperative fracture, intraoperative resuscitation, death, acute kidney failure, persistent postoperative neurological impairment until discharge, postoperative cerebral insult, wound infection requiring revision surgery, postoperative pulmonary artery embolism, severe postoperative hematoma necessitating revision surgery, and revision surgery for new biopsy. Minor complications included hypertensive crisis, uncontrollable intraoperative pain, infection with delayed wound healing, postoperative anemia, superficial wound infection, pain leading to delayed discharge, hematoma (without the need for revision), and temporary neurological impairment.

## 3. Results

**Figure 6:**
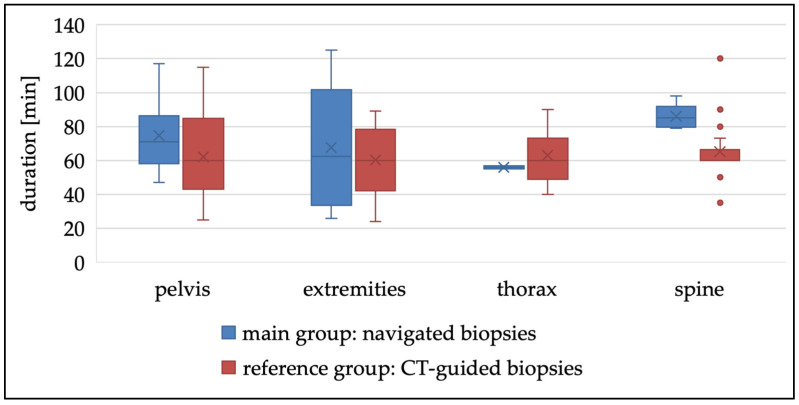
Boxplots for the duration of surgery in the different anatomical regions.

**Figure 7:**
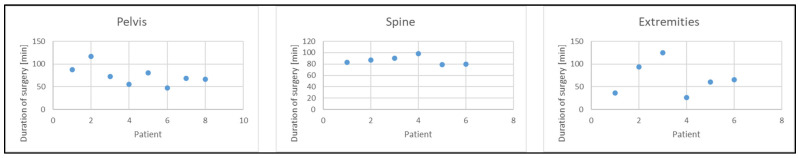
Learning curve for the duration of surgery [min] for the biopsy of tumors on the pelvis, spine, and extremities.

From 2019 to 2022, a total of 22 navigation-assisted percutaneous biopsies and 57 CT-guided biopsies of bone tumors were performed at our university clinical center. Among these, 50% (*n* = 40) were malignant, 37% (*n* = 29) benign tumors, and 13% (*n* = 10) inflammatory lesions (Table 1). In the main group, a nearly balanced gender distribution was observed with 45.5% male and 54.5% female patients. In contrast, male patients predominated in the reference group, accounting for 63% (*p* = 0.153). The mean age was 39 years (Q1: 16; Q3: 60) in the main group and significantly higher in the reference group at 63 years (Q1: 54; Q3: 74) (*p* < 0.001). In the main group, biopsies were most frequently performed on tumorous lesions located in the pelvis (*n* = 8; 36%), spine (*n* = 6; 27%), and extremities (*n* = 6; 27%). By contrast, in the reference group (CT-guided biopsies), biopsies were predominantly performed on lesions in the spine (*n* = 28; 50%) and pelvis (*n* = 17; 30%). The total radiation dose in the main group was 0.579 mSv, compared to 0.687 mSv in the reference group. The mean duration of the biopsy procedure was 69′ min in the main group, slightly longer than the 60′ min recorded in the reference group (Figure 6). The corresponding learning curve for surgeons is shown in Figure 7.

For further evaluation, the body regions of the pelvis, spine, extremities, and thorax were analyzed individually (Table 2). 

In the **pelvis**, the mean duration of surgery in the main group was 71 min, which was 18% longer than the mean of 60 min in the reference group (*p* = 0.187). The mean effective dose was 0.77 ± 0.564 mSv in the main group and 0.93 ± 1.604 mSv in the reference group (*p* = 0.464). No complications were observed in the main group, while one major complication (3.6%) occurred in the reference group. This involved bleeding from a metastasis of a renal cell carcinoma. Repeat biopsies were not required in the main group, whereas in the reference group, a second biopsy was necessary in 7% of the cases (*p* = 0.257). In 50% of cases in the main group, a subsequent surgical resection was performed in a second intervention, compared to 46% in the reference group. The histopathological result of the initial biopsy was confirmed in 100% of cases in the main group, compared to 75% in the reference group (*p* = 0.285).

In the **spine**, the mean duration of surgery in the main group was 85 min, which was 42% longer than the mean of 60 min in the reference group (*p* = 0.004). The mean effective dose was 1.398 ± 1.903 mSv in the main group and 0.718 ± 0.411 mSv in the reference group (*p* = 0.944). No complications were observed in the main group, whereas compared occurred in 11.8% of cases (*n* = 2) in the reference group, including pneumothorax and aspiration. Repeat biopsies were not required in the main group, while in the reference group, a second biopsy was necessary in 17.6% of cases (*p* = 0.270). Subsequent surgical resection was performed in 50% of cases in the main group, compared to 23.5% in the reference group. The histopathological diagnosis from the initial biopsy was confirmed in 100% of cases in the main group. In contrast, the concordance between biopsy results and the final histopathological findings was 75% in the reference group (*p* = 0.350). Regarding tumor grading, one patient in the reference group showed an upgrading of a chondrosarcoma from G1 to G2 during definitive resection following the initial CT-guided biopsy (Table 2).

In the **extremities**, the mean duration of surgery in the main group was 63 min, which was 5% longer than the mean of 60 min in the reference group (*p* = 0.712). The mean effective dose was 0.014 ± 0.03 mSv in the main group and 0.4 ± 0.51 mSv in the reference group (*p* = 0.565). No complications were observed in either the main group or the reference group. Repeat biopsies were not required in the main group, whereas a second biopsy was necessary in 40% of cases in the reference group (*p* = 0.087). Subsequent surgical resection was performed in 67% of cases in the main group, compared to 40% in the reference group. The histopathological diagnosis from the initial biopsy was confirmed in 100% of cases in both groups.

In the **thorax**, the mean duration of surgery in the main group was 56 min, which was 7% shorter than the mean of 60 min in the reference group (*p* = 0.378). The mean effective dose was 0.133 ± 0.035 mSv in the main group and 0.7 ± 0.42 mSv in the reference group (*p* = 0.142). No complications were observed in the main group. In the reference group, the intervention had to be stopped in one patient (14.3%) due to resistant pain. Repeat biopsies were not required in the main group, whereas in the reference group, a second biopsy was necessary in 2% of cases (*p* = 0.571). Subsequent surgical resection was performed in 50% of cases in the main group, whereas no resections were performed in the reference group. The histopathological diagnosis from the initial biopsy was confirmed in 100% of cases in the main group.

In 50% (*n* = 11) of the patients in the main group, PET-MRI/-CT was performed preoperatively. In contrast, only 12% (*n* = 7) of patients in the reference group underwent PET-MRI/-CT prior to the CT-guided biopsy.

Regarding the type of bone tumor or metastasis (osteolytic or osteoblastic lesion), no influence on the biopsy result was observed. There was no significant difference between the main and reference group in terms of the type of bone tumor (*p* = 0.082, Table 1). The re-biopsy rate and changes in histopathology (either in the resection specimen or the re-biopsy result) were also unaffected by the type of lesion (*p* = 0.291 and *p* = 0.686, respectively).

## 4. Discussion

The biopsy of musculoskeletal tumors, particularly of bone lesions, is a critical procedure and the decisive final step in the diagnostic algorithm, guiding subsequent multimodal therapeutic management. Incisional/open or CT-guided biopsies are the most commonly performed techniques. Open biopsies have been shown to provide increased accuracy for musculoskeletal tumors [19], reliably enabling the sampling of sufficient volume of viable tumor tissue [11,12,16]. However, open biopsies are associated with an inherently higher risk of complications, such as tumor cell dissemination, postoperative infections, and additional destabilization of the affected skeletal segment, which may already be compromised by osteolytic tumor growth [19]. These risks can be avoided by minimally invasive techniques, such as CT-guided fine or core needle biopsies. These techniques, however, pose challenges, particularly in osteoblastic tumors, as they often result in smaller tumor sample sizes, which may be insufficient for further diagnostic assessment or scientific studies such as tumor banking. Furthermore, drilling into osteoblastic lesions may affect the histopathological results or diagnosis due to potential thermal effects. 

Independently of the type of biopsy, intraoperative identification of tumor masses can be challenging, particularly when then tumors are relatively small and/or located in anatomically complex regions. This difficulty is exacerbated when only intraoperative biplanar radiographic imaging with a conventional image intensifier is available. Additionally, many tumor entities are heterogeneous, exhibiting variable intratumoral differentiation with both low- and high-grade regions within the same tumor. Targeting and biopsying the high-grade areas is crucial, as these regions are prognostically decisive and guide therapeutic decision-making. This includes the planning of subsequent modalities such as (neo-)adjuvant chemoradiotherapy or surgical excision.

Intraoperative identification of intratumoral metabolically active and high-grade regions without radiographic distinctive morphology is not feasible using classic open biopsy techniques. The identification of these high-grade areas currently relies predominantly on subjective visual correlation between contrast medium enhancement in CT/MR imaging or FDG uptake in PET imaging with fluoroscopy and the surgical site. This method carries the risk of false-negative or falsely low-grade biopsy results. Recent studies have highlighted this issue, demonstrating the high diagnostic efficacy of PET/CT-guided percutaneous needle biopsy for bone tumors. These studies report a first-time diagnostic success rate of up to 96% and an overall diagnostic success rate and sensitivity of 100% [20,21,22,23,24,25,26,27,28].

Apart from these investigations, and following extensive literature research, we identified studies focused on navigated tumor resections [29,30,31], but no studies specifically addressing surgical, minimally invasive, navigated tumor biopsies. The aim of this study, therefore, was to introduce the technique of surgical, percutaneous minimally invasive, navigated tumor biopsies and to compare it with the well-established CT-guided biopsy procedure in terms of accuracy, radiation exposure, safety, duration of the procedure, and complication rates.

The navigated biopsies were performed in both anatomically more complex regions (e.g., spinal, intrapelvic, presacral, and femoral head sites) and anatomically less complex areas (e.g., very small lesions in the proximal tibia, humerus, and sternum). In our experience, navigation-assisted, minimally invasive percutaneous biopsies are particularly beneficial when the lesion is smaller and the anatomical region is more complex—such as in cases where the tumor is near essential neurovascular structures or surrounded by thick soft tissue (e.g., biopsy tract length). In these cases, additional intraoperative orientation is particularly desirable and necessary. Furthermore, in the main group, a navigated rongeur was frequently used, whereas in the reference group, only core needles through cannula systems were used. This difference may have influenced both the quantity and representativeness of the tissue samples harvested.

The evaluation showed an accuracy and first-time success rate (diagnostic yield) of the biopsies in the main group of 100% without a need for re-biopsy. In contrast, in 14% to 40% of the cases in the reference group, a re-biopsy had to be performed. In previous studies, a wide range for the diagnostic yield of CT-guided biopsies from 68 to 98% was shown [3,4,5,6,7,8,9].

When evaluating the duration of surgery, it was observed that the average duration for CT-guided biopsies was shorter than for those in the main group. This difference is statistically significant in spinal biopsies. In spinal procedures, the reference base for navigation is not fixed using percutaneously inserted, threaded pins as in other anatomical regions (e.g., pelvis, extremities). Instead, it is placed and attached to the tip of the corresponding spinous processes of the index level using a clamp. The preparation of the processus spinosus via a mini open approach requires more time than the percutaneous insertion of pins. Additionally, the correct segmental localization and fixation of the clamp at the corresponding index level must be confirmed using an intraoperative X-ray. Despite this, the mean procedure duration still falls within the upper range of the average values for percutaneous or CT-guided biopsies reported in previous studies, ranging from 13 to 91 min [9,25,32]. However, it should be mentioned that from 2019 to 2022, the duration decreased across all groups following an initial incline, demonstrating a clear learning curve as the surgical team, including both surgeons and nurses, adapted to the new instruments, technical setup, and workflow. With increased experience, further reductions in surgery duration and radiation exposure are expected. This is also true for CT-guided biopsies, some of which were performed by residents under the supervision of a specialist, while all navigated biopsies were performed exclusively by two experienced specialists. This could also have impacted the intervention time. In contrast, only one other study from 2018 by Liu et al. reported a significantly shorter average surgery duration of 13.2 ± 4.4 min, although the accuracy was lower at 94%. This study utilized a flat-panel cone beam computed tomography virtual navigation system [9]. In our experience, surgery duration is more influenced by the preparation for the intraoperative scanning, which includes optimal patient positioning (i.e., central alignment of the target zone/scan volume in two planes), sterile draping, and the precise determination of the access path using the pointer with offset function. The adjustable offset function, combined with the orientation of the pointer angle and direction, allows the navigated pointer to percutaneously project the planned biopsy tract into the real-time 3D images of the navigation system, thereby efficiently facilitating the 3D surgical implementation of the preoperatively scheduled biopsy tract.

Compared to CT-assisted biopsies, which, according to current studies, report average radiation doses ranging from 4.3 to19.2 mSv depending on the body region examined, the average radiation dose in our study was significantly lower, ranging from 0 to 4.21 mSv [9,33]. However, the results of the calculated effective doses in the main group should be interpreted with caution due to significant deviations, in some cases up to 7000%. This discrepancy arises from factors such as the estimation of the distance between the image converter and the patient, which introduced inaccuracies in the calculations. Modern scanners tend to be less radiation-intensive, and it remains uncertain whether all available options were fully utilized during the procedure. Despite these limitations, our findings suggest that the effective dose in the main group was comparable to that in the reference group. It is important to note that the overall radiation dose to which patients were exposed should also include preoperative radiographic imaging. However, due to irregular reporting and imaging performed at external hospitals or outpatient clinics, we were unable to accurately calculate the dose of these initial radiographic studies.

Biopsy-related complications did not occur in the main group in this study. In the reference group, a total of four complications were reported, which included aspiration under general anesthesia, bleeding from a metastasis of a renal cell carcinoma, termination of the biopsy due to severe pain under local anesthesia, and a pneumothorax that required treatment with a thoracic suction drain. According to the current literature, the risk of complications from percutaneous bone biopsies can be as high as 10% [7,8,34,35]. Potential complications include postoperative infections, bleeding, nerve damage, postoperative pathological fractures, tumor seeding, and joint contamination. Needle tract seeding, in particular, has an estimated incidence of 0.003–0.009% in malignant tumors [36,37].

Tumor necrosis zones, intratumoral hematomas, and viable low- or high-grade areas are differentiated using contrast enhancement in the mandatory preoperative MRI/CT imaging. Matching the intraoperative 3D scan with the diagnostic CT and MR images allows for multiplanar visualization of the high-grade areas with maximum contrast enhancement, including soft tissue parts. This facilitated the precise and selective navigational guidance of biopsies targeting these areas. This procedure can be further refined using PET imaging, which provides essential information about the metabolically active portion of the tumor in a single examination. The maximum standardized uptake value (SUVmax) of ^18^FDG, as measured in high-grade sarcoma, has been found to correlate with mitotic count, grade [38,39], and potentially overall prognosis [40,41,42].

In this study, no significant difference was found in the re-biopsy rate or change in histopathology results between osteolytic and osteoblastic tumors. However, there was a trend towards a higher re-biopsy rate in patients with osteoblastic tumors who underwent CT-guided biopsies. The relatively small sample size in our study may account for the lack of a statistically significant difference. In the literature, a higher diagnostic yield for osteolytic lesions compared to osteoblastic lesions is frequently described [43,44,45,46,47]. One possible explanation for this is the thermal effects of the drilling procedure, which can adversely affect the quality of biopsy tissue. This issue is prevented by the use of alternative biopsy systems, such as trocar, rongeur, or punch systems, which were used in navigated biopsies in our study.

The fusion of MR imaging with PET-CT datasets combines the anatomical information from CT data with the metabolic characterization from PET, significantly improving target selection accuracy. This fusion allows for the direct biopsy of the hypermetabolic region within the bone lesions. This advantage becomes particularly evident because some PET-hyperbolic target zones may not be visible on MRI/CT scans or intraoperative fluoroscopic control. This finding is supported by Cerci et al., who demonstrated that nearly 10% of all patients undergoing PET-CT guided biopsies had areas of massively enhanced FDG uptake that did not correspond with anatomical changes seen on CT. This underscores the importance of PET/FDG uptake information in selecting representative biopsy sites.

However, several limitations must be considered. First, the technique requires advanced radiographic and computer (software/hardware) skills for the multiplanar fusion of intraoperative 3D scans with preoperative CT and MRI data, especially in the spinal and pelvic regions where reference points are limited. Additionally, technical issues with the navigation system, the C-arm, and data transfer between them may theoretically disrupt the biopsy workflow. Other limitations of this study include the small number of patients, leading to a higher statistical failure rate, and the limited comparability between groups regarding radiation exposure. Radiation data were only accurately available for the CT-assisted biopsies, while for the navigated biopsies, the radiation dose had to be determined retrospectively.

## 5. Conclusions

Based on the presented data, it can be concluded that navigation-guided biopsies, especially in anatomically complex regions, can achieve a 100% first-time diagnostic yield with comparable radiation exposure to CT-guided biopsies. While CT-guided biopsies tend to have shorter operating times, they show reduced diagnostic accuracy, a higher number of required re-biopsies, and higher complication rates. In terms of the commonly observed superior diagnostic yield in osteolytic tumors, our study did not find a significant difference, which may be attributed to the relatively small patient number or the use of navigation with different instruments. The fusion of PET/CT and MRI datasets for navigation allows a surgeon to incorporate contrast enhancement and FDG uptake into target selection, improving biopsy tract planning and enhancing the reliability of the entire procedure. However, whether this technique will truly increase diagnostic quality and reduce sampling errors, as well as the rate of complications associated with open biopsies, needs to be confirmed in prospective and randomized studies with larger patient populations.

## Figures and Tables

**Table 1:** Patient characteristics.

		All *n* = 79 (%)	Main Group (Navigation-Assisted) *n* = 22 (28%)	Reference Group (CT-Guided) *n* = 57 (72%)	*p* (χ²-Test)
**Sex**	Female	33 (41.8)	12 (54.5)	21 (36.8)	0.153
	Male	46 (58.2)	10 (45.5)	36 (63.2)	
**Age in years** mean (min-max)		55.8 (9–88)	39 (9–84)	63 (17–88)	**<0.001** *
**Localization**	Pelvis	36 (45.6)	8 (36.4)	28 (49.1)	0.200
	Spine	23 (29.1)	6 (27.3)	17 (29.8)	
	Extremities	11 (13.9)	6 (27.3)	5 (8.8)	
	Thorax	9 (11.4)	2 (9.0)	7 (12.3)	
**Type of bone lesion**	Osteolytic	48 (60.8)	16 (72.7)	32 (56.1)	0.082
	Osteoblastic	20 (25.3)	6 (27.3)	14 (24.6)	
	Mixed	11 (13.9)	-	11 (19.3)	
**Histopathology**	Chondrosarcoma	8 (10.1)	4 (18.2)	4 (7.0)	-
	Ewing’s sarcoma	3 (3.8)	2 (9.1)	1 (1.8)	
	Chordoma	2 (2.5)	1 (4.5)	1 (1.8)	
	Rhabdomyosarcoma	1 (1.3)	1 (4.5)	-	
	Myxoid liposarcoma	1 (1.3)	-	1 (1.8)	
	Leiomyosarcoma	1 (1.3)	-	1 (1.8)	
	Myeloma	5 (6.3)	1 (4.5)	4 (7.0)	
	Lymphoma	1 (1.3)	-	1 (1.8)	
	Extramedullary AML ^1^	1 (1.3)	-	1 (1.8)	
	Breast cancer	7 (8.9)	3 (13.6)	4 (7.0)	
	Bronchial carcinoma	1 (1.3)	-	1 (1.8)	
	Pancreatic carcinoma	1 (1.3)	-	1 (1.8)	
	Prostate carcinoma	1 (1.3)	-	1 (1.8)	
	Renal cell carcinoma	3 (3.8)	-	3 (5.3)	
	Urinary bladder carcinoma	1 (1.3)	-	1 (1.8)	
	Tongue carcinoma	1 (1.3)	-	1 (1.8)	
	Squamous cell carcinoma (skin)	1 (1.3)	-	1 (1.8)	
	Neuroendocrine carcinoma	1 (1.3)	-	1 (1.8)	
	Aneurysmatic bone cyst	2 (2.5)	2 (9.1)	-	
	Giant cell tumor	1 (1.3)	-	1 (1.8)	
	Osteoblastoma	2 (2.5)	2 (9.1)	-	
	Hemangioma	2 (2.5)	1 (4.5)	1 (1.8)	
	Mb. Paget	2 (2.5)	-	2 (3.5)	
	Langerhans cell histiocytosis	1 (1.3)	-	1 (1.8)	
	Osteomyelitis	9 (11.4)	4 (18.2)	5 (8.8)	
	Tuberculosis	2 (2.5)	1 (4.5)	1 (1.8)	
	Other (reactive lesions, fibrosis, osteoradionecrosis)	18 (22.8)	-	18 (31.6)	

* Significant results in the 95% confidence interval. ^1^ AML: acute myeloid leukemia.

**Table 2:** Results of main group and reference group.

		All *n* = 79 (%)	Main Group (Navigation-Assisted) *n* = 22 (28%)	Reference Group (CT-Guided) *n* = 57 (72%)	*p* (χ²-Test)
**Duration (min.)** (mean)	Pelvis	66	71	60	0.187
	Spine	66.4	85	60	0.004 *
	Extremities	65.1	63	60	0.712
	Thorax	65.9	56	60	0.378
**Effective dose (mSv)** (mean)	Pelvis	0.92	0.77	0.93	0.464
	Spine	1.2	1.398	0.718	0.944
	Extremities	0.25	0.014	0.4	0.565
	Thorax	0.4	0.133	0.7	0.142
**Complications**	Pelvis	3 (3.8)	-	1 (3.6)	0.588
	Spine	4 (5.1)	-	2 (11.8)	0.379
	Extremities	-	-	-	-
	Thorax	-	-	1 (14.3)	-
**Re-biopsy necessary**	All	9 (11.4)	-	9 (15.8)	-
	Pelvis	4/36 (11.1)	-	4/28 (14.3)	0.257
	Spine	2/23 (8.7)	-	2/17 (11.8)	0.270
	Extremities	2/11 (18.2)	-	2/5 (40.0)	0.087
	Thorax	1/9 (11.1)	-	1/7 (14.3)	0.571
**Resection performed**	All	31 (39.2)	12 (54.5)	19 (33.3)	-
	Pelvis	17/36 (47.2)	4/8 (50.0)	13/28 (46.4)	-
	Spine	7/23 (30.4)	3/6 (50.0)	4/17 (23.5)	-
	Extremities	6/11 (54.5)	4/6 (66.7)	2/5 (40.0)	-
	Thorax	1/9 (11.1)	1/2 (50.0)	-	-
**Histopathology confirmed**	No changes	26/31 (83.9)	16/16 (100)	10/15 (66.7)	0.018 *
	Different result	5/31 (16.1)	-	5/15 (33.3)	
**Grading confirmed** (Sarcoma only)	No changes	11/12 (91.7)	8/8 (100)	3/4 (75.0)	0.333
	Upgrading	1/12 (8.3)	-	1/4 (25.0)	

* Significant results in the 95% confidence interval.

## Data Availability

All data obtained and analyzed for this clinical study are available from the corresponding author upon reasonable request.
